# Dissociable effects of medication on visual–vestibular brain excitability by visual motion stimuli in episodic ataxia type 2

**DOI:** 10.1093/braincomms/fcaf400

**Published:** 2025-10-24

**Authors:** Janina von der Gablentz, Andreas Sprenger, Nina Overbeeke, Dagmar Timmann, Norbert Brüggemann, Christoph Helmchen

**Affiliations:** Department of Neurology, University Hospital Schleswig-Holstein, 23538 Lübeck, Germany; Department of Neurology, University Hospital Schleswig-Holstein, 23538 Lübeck, Germany; Center of Brain, Behavior and Metabolism (CBBM), University of Lübeck, 23562 Lübeck, Germany; Institute of Psychology, University Lübeck, 23562 Lübeck, Germany; Department of Neurology, University Hospital Schleswig-Holstein, 23538 Lübeck, Germany; Department of Neurology and Center for Translational Neuro- and Behavioral Sciences, University of Duisburg-Essen, 45147 Essen, Germany; Department of Neurology, University Hospital Schleswig-Holstein, 23538 Lübeck, Germany; Center of Brain, Behavior and Metabolism (CBBM), University of Lübeck, 23562 Lübeck, Germany; Department of Neurology, University Hospital Schleswig-Holstein, 23538 Lübeck, Germany; Center of Brain, Behavior and Metabolism (CBBM), University of Lübeck, 23562 Lübeck, Germany

**Keywords:** episodic ataxia type 2, fMRI, visual stimulation, visual excitability, visual–vestibular interaction

## Abstract

The clinical hallmark of episodic ataxia type 2 (EA2) consists of episodes of recurrent severe vestibulo-cerebellar dysfunction, characterized by marked postural unsteadiness and oscillopsia. Triggering factors of EA2 attacks, such as physical exertion and sensory stimulation, the high comorbidity with migraine, and the increased risk of epilepsy in EA2 suggest abnormal brain excitability. To investigate this, we assessed brain excitability in response to visual (checkerboard) and visual motion (optic flow) stimuli using interictal functional magnetic resonance imaging. Visual stimulation elicited strong bilateral neural activity in the primary visual cortex (V1–V3) and in motion-sensitive visual areas (V5) in 21 EA2 patients and 21 age-matched healthy participants (HP). Compared to HP, EA2 patients revealed decreased activity in the primary visual cortex (V1), cerebellar Crus I and II and caudal vermis but increased activation of multisensory vestibular processing areas (posterior insula, superior temporal and supramarginal gyrus, inferior parietal lobe). Interestingly, the abnormal excitability in the vestibular processing cortex areas was primarily found in patients without medication (4-aminopyridine, acetazolamide) but hardly seen in patients on medication. Our findings in treatment-naïve patients reflect disease-inherent changes in visual cortical excitability in EA2, which may be reversible through anti-episodic medication. As excitability by visual motion stimuli in multi-sensory vestibular processing cortical areas was largely found in patients on medication it may also indicate an inhibitory effect on the physiological reciprocal inhibitory visual–vestibular interaction as a multisensory mechanism for self-motion perception: the annoying oscillopsia of EA2 patients is counterbalanced by decreased visual cortex activity and hence smaller inhibition of the vestibular cortex.

## Teaser

Interictal visual functional MRI in patients with episodic ataxia type 2 showed reduced activity in visual and cerebellar regions, but increased excitability in vestibular areas. These changes appeared mainly in unmedicated patients, suggesting disease-related cortical alterations. Treatment with 4-aminopyridine or acetazolamide may normalize brain excitability and counteract these functional abnormalities.

## Graphical Abstract

**Graphical Abstract:**
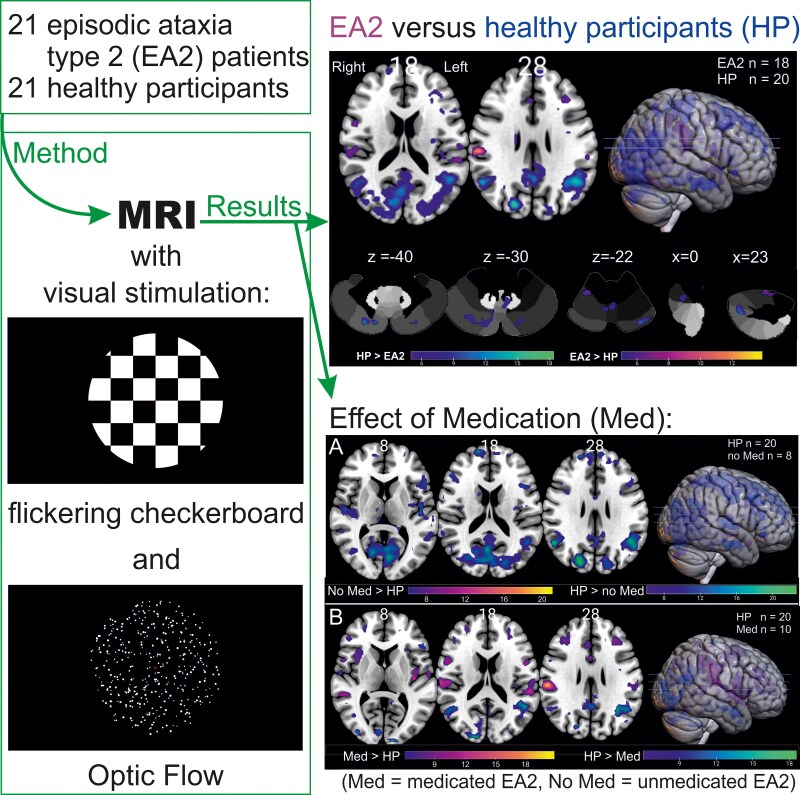

## Introduction

Patients with episodic ataxia type 2 (EA2) belong to the group of cerebellar degenerative diseases with slowly progressive symptoms and signs of cerebellar dysfunction.^1–6^ Genetically, this autosomal-dominant disorder shows monoallelic disease-causing variants in the *CACNA1A* gene on chromosome 19p13 which encode a voltage-gated subunit of the neuronal Cav2.1 P/Q-type calcium channel.^3^

In addition, the clinical hallmark of EA2 are circumscribed episodes of recurrent severe vestibulo-cerebellar dysfunction: gross postural unsteadiness, limb ataxia, slurred speech and oscillopsia lasting for several hours.^4,7,8^ These attacks are often provoked by emotional distress, alcohol or caffeine consumption, physical exertion and various sensory (e.g. vestibular, visual) stimulation suggesting abnormal brain excitability.^9^ EA2 patients show an increased risk for epilepsy^9^ and altered cortical excitability has been demonstrated by spontaneous interictal epileptic discharges observed in EEG recordings,^10^ and by transcranial magnetic stimulation.^11^ Altered cortical sensory excitability is a hallmark of migraine. Many EA2 patients suffer from comorbid migraine. In line with this, neighbouring *CACNA1A* mutations are associated not only with familial hemiplegic migraine^12^ but also with therapy-refractory epilepsy, as seen in patients with developmental epileptic encephalopathy.^13^ Additionally SCA6, a progressive ataxia sometimes associated with migraine, is also caused by mutation in the *CACNA1A* gene.^14^ Recently, a neural model of haploinsufficiency of the *CACNA1A* gene exhibited increased intrinsic excitability via diminished potassium channel function.^15^ The vestibulo-cerebellar signs in EA2 can be linked to a loss- or a gain-of-function changing the presynaptic excitability in the cerebellar Purkinje cells.^3^ A related mechanism has been identified in spinocerebellar ataxia (SCA) type 27B with late-onset progressive cerebellar degeneration and episodic attacks in many but not all patients, usually at disease manifestation. SCA27B is associated with an intronic trinucleotide repeat in *FGF14* encoding fibroblast growth factor 14, which is important in regulating the distribution of voltage-gated sodium channels in cerebellar Purkinje and granule cells.^5,16^

Accordingly, 4-aminopyridine (4-AP) reduces the number and severity of attacks in EA2 and SCA27B by lowering the abnormal excitability of the cerebellar Purkinje cells.^6,17,18^ Thus, studying EAs has illuminated previously unrecognized but important roles of ion channels and transporters in brain function with shared mechanisms underlying cerebellar ataxia, migraine and epilepsy.^5^ However, it has not been tested yet how sensory stimuli affect the brain’s excitability using functional imaging techniques.

Imaging studies in the interictal interval of patients with *CACNA1A* mutations (EA2, familial hemiplegic migraine, SCA6) revealed progressive cerebellar degeneration, in particular of the vestibulo-cerebellum,^2,19,20^ leading to cerebellar atrophy.^21^ Until now, it is not known how these recurrent episodes of visual-vestibular dysfunction affect neurodegeneration in the brain and its cortical excitability to sensory stimulation of EA2 patients.

As a consequence of vestibulo-cerebellar degeneration, EA2 patients suffer from gaze holding deficit, often significant downbeat nystagmus leading to vertical interictal oscillopsia with impaired visual acuity. Physiological strategies to counterbalance oscillopsia are alterations of visual cortex excitability, in particular motion sensitivity. This functional compensation reduces the annoying aspects of involuntary eye oscillations but alters visual sensitivity for moving targets in the visual surrounding. Visual motion sensitivity has been found to be reduced in patients with cerebellar downbeat nystagmus^22^ and bilateral vestibular hypofunction.^23^ In accordance, patients with involuntary ocular oscillations show a deactivation of the visual cortex 24.

We hypothesized that (i) cortical responses to visual motion stimuli are reduced in EA2 patients and (ii) this reduction can be modulated by pharmacological treatment with acetazolamide or 4-AP, both established therapies for the prevention of EA2 attacks. We used two stimuli (checkerboard and optic flow) designed to stimulate not only the primary visual cortex but also motion-sensitive higher visual cortex areas (MT/V5). Based on the physiological reciprocal inhibitory interaction of the visual and vestibular cortex,^24^ we expected increased vestibular cortex activation resulting from the reduced visual inhibition of multisensory vestibular cortex areas (e.g. posterior insula).

## Materials and methods

The study was approved by the Ethics Committee of the University of Lübeck (#16-068) and conducted in accordance with the Declaration of Helsinki and the World Medical Association’s Code of Ethics. Following written informed consent, we enrolled 21 patients with previously diagnosed EA2 and confirmed pathogenic heterozygous mutations in the *CACNA1A* gene. In addition, a control group of 21 healthy participants (HP), matched for age and sex, was included.

Clinical exclusion criteria included dementia, major depression, personality disorders, continuous use of sedative drugs (benzodiazepine, narcotics, barbiturate, neuroleptics, opioids, antidepressant agents, antiepileptic agents) or alcohol consumption, contraindications for MRI, or structural brain lesions. MRI-specific exclusion criteria included structural CNS lesions (e.g. Arnold-Chiari malformation, Wernicke´s encephalopathy).

All HP had normal or corrected-to-normal vision and a normal peripheral vestibular function, based on the medical history and a neurological examination. Furthermore, HP with a history of migraine were only included if they had a negative genetic test for *CACNA1A* (two family members of EA2 patients).

All participants underwent a comprehensive review of medical history and a neurological examination. Cerebellar signs were assessed using the Scale for the Assessment of Rating of ataxia (SARA).^25^ Non-ataxia symptoms were evaluated with the Inventory of Non-Ataxia Symptoms (INAS), specifically developed for ataxia patients.^26^ Neuro-otological assessments were scored using the Clinical Vestibular Score (CVS)^27^ and cognitive impairment was evaluated by the Montreal Cognitive Assessment (MoCA).^28^ To evaluate finger dexterity, despite its limited specificity for cerebellar ataxia, the 9-Hole Peg Test (NHPT)^29^ was administered. Handedness was determined objectively using Edinburgh Handedness Inventory.^30^ Finally, patients rated their disease-related impairment using the Dizziness Handicap Inventory (DHI).^31^ In this score larger values indicate increased vestibular-induced subjective disability. Additionally, all participants underwent neuro-otological examinations, including quantitative head impulse test (qHIT) and subjective visual vertical (SVV).

### Statistical analysis of clinical data

Statistical analyses were performed using SPSS (Version 22; IBM Corporation, Armonk, NY). To examine associations between demographic data and groups (EA2 versus HP), we used cross-tabulations. For clinical data, normality was assessed using the Shapiro–Wilk test. Group differences in VOR and SVV values were analysed using *t*-tests. For non-normally distributed data, Mann–Whitney U test were applied to compare clinical scores (SARA, INAS, CVS, MoCA, 9-Hole Peg Test) between the groups.

### Visual stimulation

For visual stimulation inside the MRI scanner, participants viewed a black screen (36 × 70 cm at a distance of 134 cm) through a mirror. The stimulation consisted of three conditions: (i) Rest: a red fixation dot (0.15°) positioned at the centre of the screen; (ii) Checkerboard (CB): a checkerboard in a 12° sized annular region composed of 2°-sized black-and-white squares, which changed colours every 60 ms. The red fixation dot (0.15°) was presented at the centre of the CB; (iii) Optic Flow (OF): 2000 white dots (0.2° in diameter) on a black screen, moving outward from the centre at a speed of 7°/s within an annular region spanning 1° to 12°, with the fixation dot (0.15°) in the centre. All three conditions (CB, OF, Rest) were presented in a pseudo-randomized order for durations of 10 s (Rest) or 12 s (CB, OF), repeated six to nine times. During MRI data acquisition, eye movements were recorded using the video-based Eye Tracker EyeLink 1000 Plus (software version 5.50, SR Research Ltd., Ottawa, Canada) with a sampling frequency of 1000 Hz. Horizontal and vertical eye positions were analysed offline using Matlab® (R2022b, The Mathworks Inc., Natick, MA, USA). This data was used to monitor fixation.

### Image acquisition

We acquired structural and functional MRI at a 64-channel head-coil mounted 3T Siemens Magnetom Skyra scanner at the Center of Brain, Behaviour and Metabolism at the University of Lübeck. Anatomical T1-weighted images were acquired using an T1-weighted MP-RAGE sequence (repetition time 1900 ms, echo time 2.44 ms, inversion time 900 ms, flip angle 9°, 1 × 1 × 1 mm³ resolution; matrix 256 × 256 mm, 192 sagittal slices).

Functional T2*-weighted images were acquired using an echo-planar imaging sequence sensitive to blood oxygen level dependent (BOLD) contrast. The acquisition parameters were as follows: repetition time = 1220 ms; echo time = 31 ms; flip angle = 70°; 60 transversal slices; 477 volumes; voxel size 2.5 × 2.5 × 2.5 mm, simultaneous multislice acceleration factor: 4. To minimize noise and head motions we used ear plugs and ear pads (Multipad ear, Pearltec Technology AG, Schlieren/CH).

### Preprocessing and MRI data analysis

Preprocessing and subsequent image analysis were performed using Statistical Parametric Mapping software (SPM12b, Wellcome Trust Centre for Neuroimaging, London, UK; http://www.fil.ion.ucl.ac.uk/spm) implemented in Matlab® 2022B (MathWorks, Natick, MA). Preprocessing steps included slice-timing correction, motion correction through rigid body spatial realignment to the mean functional image of each dataset and normalization to the Montreal Neurological Institute (MNI) template. The images were resampled to a 2.5 × 2.5 × 2.5 mm³ voxel size and smoothed with a 6 mm full-widths half-maximum (FWHM) Gaussian kernel. We assessed individual head motion and excluded trials where maximum motion in any XYZ direction exceeded 2.5 mm. As a result, three patients and one HP were excluded, along with trials from 9 patients and 4 HPs. Statistical parametric maps were computed for each participant on a voxel-by-voxel basis using a general linear model, with one regressor for each of the three conditions. The following contrasts were defined: OF versus Rest, CB versus Rest, OF versus CB, CB versus OF. To investigate the effect of visual stimulation (Rest, OF, CB) on the two groups (EA2, HP) we performed a 3 × 2 flexible factorial design as outlined in.^32^ All results were assessed for cluster-wise significance with a cluster-defining threshold of *P* = 0.05, corrected for family-wise error (FWE), and a minimum cluster size of 10. Anatomical regions were identified using the Automated Anatomical Labelling (AAL) Atlas.^33^

In addition to the whole brain analysis, we conducted a region of interest (ROI) analysis, focusing on regions specified a priori, including both visual and vestibular areas. For ROI definition, we used the eBrains Siibra tool with the Multilevel Human Atlas, the ICBM 2009c template and the Jülich-Brain v3.1 parcellation (https://atlases.ebrains.eu/).^34^ The following ROIs were defined for both sides: V1 (Area hOc1), V2 (Area hOc2), V3 (Area hOc3d), V5/MT + (Area hOc5), PF (IPL = inferior parietal lobule), Cingulate sulcus (CSv) (Area 5 M), parietal operculum (OP1-4). Additionally, we created ROIs in the visual area of the cingulate cortex on both sides, in the Uvula, in the vermian lobule VI and VII, and on both sides in the flocculus (lobule X), dorsal dentate nucleus and the fastigial nucleus using MarsBar^35^ to define a 5 mm radius sphere around coordinates reported by Ruehl, Flanagin, Ophey, Raiser, Seiderer, Ertl, Conrad and Zu Eulenburg.^36^ Whole-brain results and ROIs were visualized using MRIcroGL.^37^

Using the contrast values from each subject within each ROI we analysed the group difference and correlated the results with clinical parameters (age, age at disease onset, disease duration, number of attacks per month, duration of attacks in hours and attack intensity, SARA, CVS, DHI) using the Spearman rho coefficient. Due to the exploratory nature of the analysis, we report the uncorrected *P*-values. To distinguish differences between patients more severely affected during the interval and those with fewer cerebellar signs during the interval, we divided the patients into two groups (split-half analysis) based on their interictal SARA scores (<4 points and ≥4 points). A *t*-test was then calculated to find differences of the contrast values between these patient groups in each ROI. Results were expected as significant at a *P*-value < 0.05.

## Results

### Clinical data

Demographic data, clinical history and results of the neurological and neuro-otological examination, including all assessed scores, are presented in Table 1 for 18 EA2 patients and 20 HP (3 patients and 1 HP had to be excluded due to head motion in MRI). The two groups showed no significant differences in age, sex or handedness (94.4% right-handed among patients, 95% among HP). Almost all EA2 patients reported experiencing ataxia attacks with dizziness and unsteadiness, with an average frequency of 5.03 ± 6.84 attacks per month lasting an average of 3.55 ± 3.53 h, and an intensity of 7.24 ± 2.08 on a scale of 0 to 10. Average disease duration was 10.8 ± 6.0. The disease onset was defined as the first remembered attack or symptom. Common triggers included physical exertion (88%), stress (88%), caffeine (35.3%) or alcohol (35.2%). Of the patients, 33.3% were on acetazolamide (250–500 mg/day), 22% on 4-AP (Fampyra^TM^ 20 mg/day), while 44% where not on any medication. Some patients without continuous medication did not want to try a medication, others discontinued the therapy due to adverse effects or a lack of efficacy.

**Table 1:** Demographic and clinical characteristics of patients and healthy participants

	EA2 patients	Healthy participants	Level of significance
Age (yrs)	41.8 ± 13.4	41.3 ± 14.4	n.s.
Sex (n female/male)	7/11	9/11	n.s.
Age at diagnosis (yrs)	34.1 ± 16.0	**-**	**-**
Disease duration (yrs)	10.8 ± 6.0	**-**	**-**
Attacks/month	5.0 ± 6.4		
attack duration (h)	3.55 ± 3.53		
SARA	4.03 ± 3.46	0.05 ± 0.22	0.00
INAS	5.67 ± 3.09	1.10 ± 1.65	0.00
DHI	38.89 ± 26.73		
CVS	4.5 ± 4.41		
MoCa	25.00 ± 3.57	28.45 ± 2.09	0.00
NHPT (s), dominant	20.68 ± 2.85	17.49 ± 1.80	0.00
NHPT (s), n.d.	21.24 ± 3.3	18.51 ± 1.90	0.00
SVV, dynamic	−0.33 ± 2.2	−0.47 ± 1.27	n.s.
SVV, static	0.42 ± 1.29	−0.40 ± 1.14	n.s.
vHIT right	0.84 ± 0.25	0.99 ± 0.13	0.048
vHIT left	0.9 ± 0.29	1.1 ± 0.11	0.01

Between the ataxic episodes with unsteadiness, clinical examination revealed gaze-evoked nystagmus in 50%, head-shaking nystagmus in 33% and abnormal smooth pursuit in horizontal and vertical direction in 78%. Most patients (83%) demonstrated gait and stance ataxia, and limb ataxia was observed in up to 61%. Significant differences were noted, as anticipated, in the SARA and INAS (oculomotor signs in EA2 patients) scores as well as in the MoCA score between EA2 patients and HP. There were no differences in the SVV, though patients performed significantly worse on the NHPT and the qHIT (see **Table 1**). Clinical information of subgroup analyses (SARA ≥ 4 versus SARA < 4 and patients with versus without medication) are shown in Supplementary Tables 4 and 5.

### Whole-brain analysis

**Figure 1:**
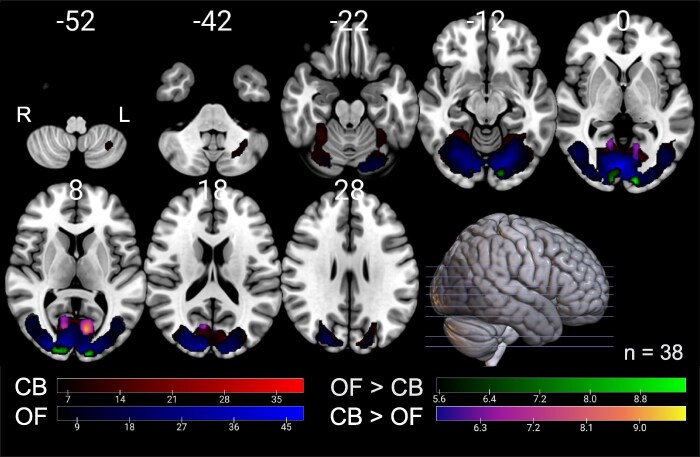
**Activations during OF and during CB stimulation.** Areas with significant BOLD activation during visual stimulation in all subjects (patients and HP) are colour-coded: red for CB stimulation and blue for OF stimulation. Green areas indicate significantly greater activation during OF stimulation compared to CB stimulation, while areas with more activation during CB stimulation are shown in plasma (purple to yellow). We avoided orange colours. The colour gradient reflects the *t*-value, beginning at the threshold of FEW-corrected *P* ≤ 0.05. MNI *z*-coordinates for each transverse section are provided above. L = left; R = right, *n* = number of subjects.

Using whole-brain analysis in all participants (EA2 and healthy persons), we identified significant activation (FWE corrected *P* < 0.05) in primary visual areas (V1, V2, V3) and thalamus, hippocampus, dorsal dentate nucleus during both OF and CB stimulation, compared to the resting condition (see Fig. 1 and Supplementary Table 2). Contrasting OF and CB stimulation, OF elicited significantly stronger activation in cuneus and lingual gyrus (V1) as well as in V2 and V3, whereas CB stimulation showed significantly more activation within the calcarine visual sulcus (V1) (see Fig. 1 and Supplementary Table 2).

**Figure 2:**
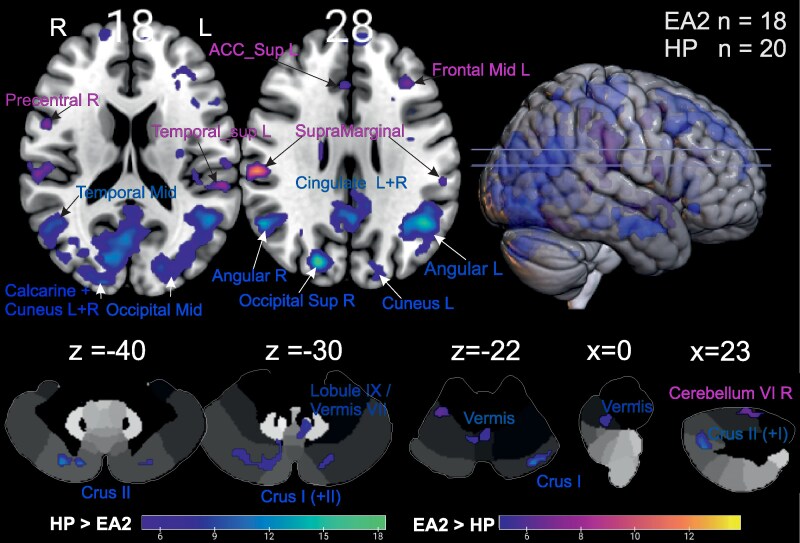
**Differences between EA2 patients and HP in whole-brain activation.** Areas with significantly greater BOLD activation in one group are color-coded: plasma colours (purple to yellow) indicate higher activation in EA2 patients than in HP, while cold colours (blue to green) indicates higher activation in HP compared to EA2 patients. The colour gradient reflects the *t*-value, beginning at the threshold of FWE-corrected *P* ≤ 0.05. The upper row displays activations on a standard MNI template. The lower row shows BOLD activation in the cerebellum, presented on an MNI cerebellar Atlas by.^38,39^ If not stated otherwise, *z*-coordinates are shown above each slice. L = left, R = right, Sup = superior, ACC = anterior cingulate cortex, *n* = number of subjects.

Group comparison revealed significantly greater activation in EA2 patients in the supramarginal gyrus bilaterally, the left middle frontal gyrus, the inferior parietal gyrus and the superior temporal gyrus bilaterally as well as in the right cerebellar hemisphere of lobule VI, the hippocampus, the thalamus, the cingulate gyrus, and the anterior cingulum (see Fig. 2 and Supplementary Table 1). HP exhibited greater activation in large parts of the primary visual regions, including the middle occipital lobe, the cuneus, the calcarine sulcus (V1/V2), and the middle temporal gyrus (MT/V5) bilaterally. Additionally, the cingulate and fusiform gyrus, the angular gyrus as well as the supplementary motor area bilaterally, the medial and superior frontal gyrus bilaterally, and cerebellar regions (left Crus I, Crus II bilaterally, and the vermal lobules VI/VII and parts of IX) were significantly more activated bilaterally in HP.

### ROI analysis

**Figure 3:**
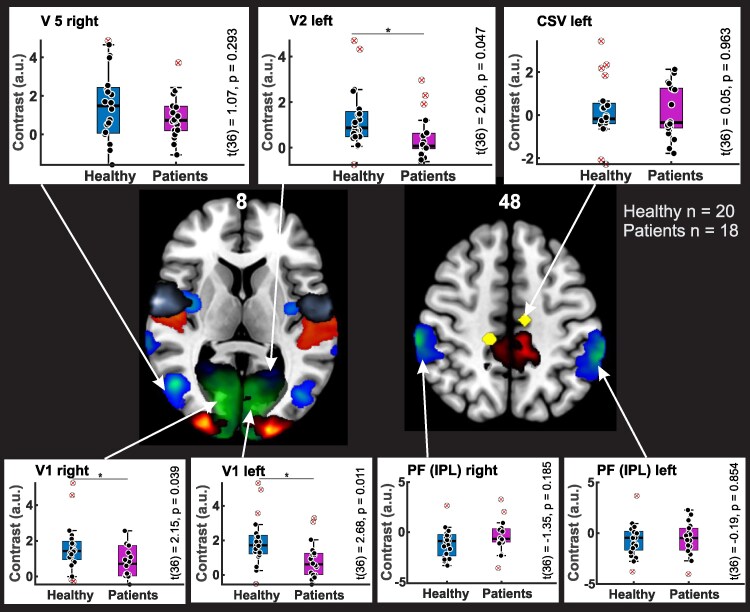
**Activation differences between EA2 patients and HP in distinct ROIs.** All ROIs are colour-coded on an MNI template. Differences between specific ROIs are presented as boxplots, with individual data points shown in black. The interquartile range for controls is represented in blue, and patients in purple. *t*-Values and *P*-values are displayed on the right side of each boxplot, with significant results (*P* ≤ 0.05) marked with an asterisk (*). ROI colour-coding: V1 = green, V2 = blue, V3 = red-yellow (warm), V5 = blue to green (winter), Area PF (IPL = inferior parietal lobule) = blue to green (winter), Cingulate Sulcus visual Area (CSv) = yellow, Area 5 M (superior parietal lobule) = red (red/black), OP1 = red, OP3 = blue, OP4 = grey (bone). ROIs not shown due to slice selection: OP2, Cerebellar ROIs. *n* = number of subjects.

The ROI analysis was conducted for visual and vestibular areas bilaterally: V1, V2, V3, V5/MT+, PF, CSv, parietal operculum (OP1-4), cingulate cortex, uvula, dorsal dentate nucleus, vermian lobule VI and VII, flocculus, fastigial nucleus. There was larger activation of the visual cortex in HP, specifically in V1 bilaterally for OF versus Rest (right: *t*(36) = 2.15, *P* = 0.039, left: *t*(36) = 2.68, *P* = 0.011) and CB versus Rest (right: *t*(36) = 2.11, *P* = 0.042, left: *t*(36) = 2.34, *P* = 0.025) as well as in the left V2 area for OF versus Rest (*t*(36) = 2.06, *P* = 0.047) (see Fig. 3). This larger activation in V5 failed statistical significance in the ROI analysis. There was no difference in visually evoked activity between EA2 patients and HP in the inferior IPL and in the visual area of the CSv as well as in the OP1-4, in visual area V3 and in the uvula.

### Correlation of clinical data with ROI activity

**Figure 4:**
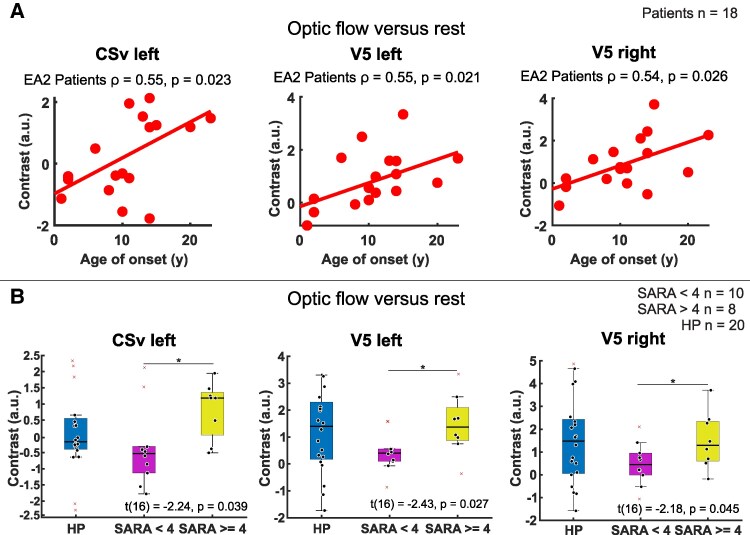
**Significant correlation with age of onset and group differences between stronger versus less affected patients.** (**A**) Shows significant Spearman’s rho correlations between the ‘age of onset’ in years (y) and the contrast values within the left cingulate visual area (CSv) and the V5 area bilaterally in EA2 patients. Corresponding *r*- and *P*-values are indicated above. (**B**) Displays boxplots with significant differences in BOLD activation (contrast values) among less affected patients (SARA < 4, purple), and more affected patients (SARA ≥ 4, yellow) within the left CSv and bilaterally in the V5 area. Statistical comparison was performed only between these two groups using *t*-test. The values of the healthy participants (HP, blue) are shown for reference only, without statistical testing. Relevant *t*- and *P*-values for patients are shown. *n* = number of subjects.

No meaningful correlations were identified between clinical parameters of patients (including disease duration, attack intensity, attack duration, number of attacks per month, CVS, INAS, and SARA) and differences in neural activity across conditions (OF, CB), within each group, or between groups. However, a significant correlation was observed for the *age at onset* in EA2 patients with contrast values of optic flow versus rest in the left CSv (*r* = 0.55, *P* = 0.023), and bilaterally in V5 (left: *r* = 0.55, *P* = 0.021; right: *r* = 0.54, *P* = 0.026) (see Fig. 4A). Specifically, neural activity in response to visual motion stimuli increased with later age at onset of EA2-related symptoms. Noticeably, visual motion-evoked activity in bilateral V5 increased with the age of HP (left *r* = 0.57, *P* = 0.008, right *r* = 0.47, *P* = 0.035) during OF (versus Rest) and in the right V5 (*r* = 0.45, *P* = 0.044) and in the left PF (*r* = 0.54, *P* = 0.013) during CB (versus Rest) (see Supplementary Fig. 1). In contrast, this correlation with age was not found in EA2 patients. Additional results regarding the correlation with clinical data are provided in the Supplementary material. For the oculomotor component of the INAS, we found a significant correlation with the left Visual Cortex Area V1 (OF *P* = 0.03; CB *P* = 0.04) and the right Area V1 (OF *P* = 0.024; CB *P* = 0.032).

#### Split-half analyses (SARA)

Patients with a higher SARA score (SARA ≥ 4) showed significantly higher contrast values in the left CSv (*t*(16) = −2.24, *P* = 0.039) and bilaterally in V5 (left: *t*(16) = −2.43, *P* = 0.027; right: *t*(16)=−2.18, *P* = 0.045) compared to patients with a SARA-score < 4 during optic flow stimulation (see Fig. 4B).

### Effect of medication

**Figure 5:**
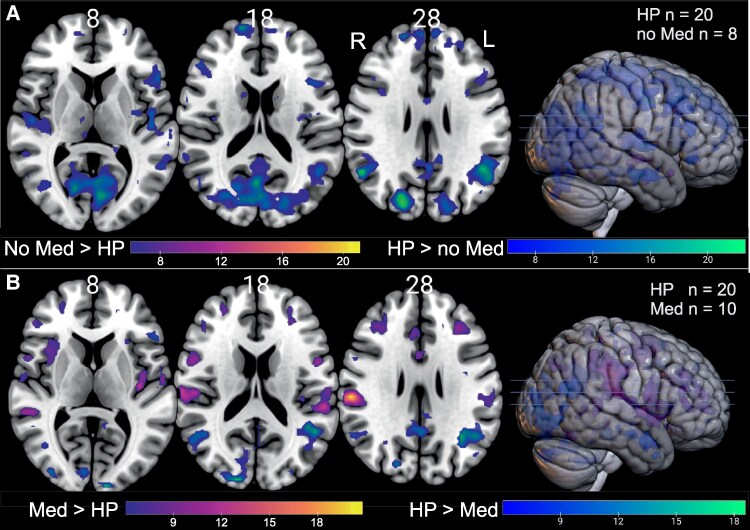
**Activation differences between HP and EA2 patients without (A) or with medication (B).** Part **A** illustrates significantly higher BOLD activation of HP represented in blue/green, compared to EA2 patients without medication (No Med), who exhibit minimal greater activation (shown in purple/yellow). Part **B** highlights the significantly higher BOLD activation of EA2 patients taking medication (Med)—specifically Acetazolamide or fampridine—displayed in purple/yellow. In contrast, areas of greater activation in HP remain depicted in blue/green. The colour gradient corresponds to the *t*-value, starting at a threshold of FEW-corrected *P* ≤ 0.05. MNI *z*-coordinates for each transverse section are indicated above the slices. L = left; R = right; n 0 number of subjects.

There was a strong effect of medication on visually evoked activity. Subgroups comparison of patients with (*n* = 10) and without (*n* = 8) medication versus HP revealed lower activation in the primary visual cortex of patients *without* medication (Fig. 5A, Supplementary Table 3). EA2 patients *on* medication showed activation in the visual cortex almost indistinguishable from HP (Fig. 5B) but larger activation in multisensory vestibular cortex (Fig. 5B, Supplementary Table 3).

## Discussion

EA2 is a primarily cerebellar disease that typically presents with cerebellar attacks of unsteadiness indicating changes in neuronal excitability. In line with this hypothesis, our study using visual stimuli disclosed altered excitability not only in the cerebellum but impressively in cortical visual and multimodal vestibular processing areas in EA2 patients. While EA2 patients showed lower excitability in the visual cortex (V1, V2, V5), cerebellar Crus I and II and caudal vermis, it was larger in multisensory vestibular processing areas (posterior insula, superior temporal and supramarginal gyrus, inferior parietal lobe) compared to healthy control subjects (see Fig. 2). Interestingly, this abnormal excitability was largely found in unmedicated EA2 patients, as the effects were hardly found in medicated patients. This could provide a new basis for a controlled therapeutic trial (4-AP, acetazolamide) as our findings in treatment-naïve patients reflect disease-inherent changes of visual cortical excitability in EA2 which can probably be reversed by anti-episodic medication.

Our two visual stimuli (CB, OF) were suitable for testing cortical excitability as they elicited strong bilateral neural activity in the primary visual cortex as well as secondary visual cortex areas supplied by the dorsal stream conveying motion-related information up to MT/V5, in patients and HP. There was a substantial portion of overlapping activation in the medial aspects of the lingual gyrus and cuneus. In line with previous reports,^40^ CB (versus OF) stimulation revealed stronger activation in the calcarine visual cortex, while OF showed stronger activation in the cuneus and lingual gyrus when contrasted with CB. The strong difference between patients with versus without medication on the visual and vestibular cortical excitability implies an explanation for the effect of attack-reducing medication.

### Excitability in the visual cortex

Group contrasts revealed reduced excitability of large parts of the primary visual cortex (V1, V2) of EA2 patients while no overall group differences were observed in V5/MT or the CSv.

While V1 and V2 respond to static visual stimuli (CB), neural activity in higher visual cortical areas, particularly V5/MT, is related to egomotion-inducing visual motion. Accordingly, the extrastriate middle temporal visual area V5/MT contains neurons that are active during smooth pursuing eye movements of slowly moving objects.^41^ V5/MT is involved in discriminating self- from object-motion, especially during the recognition of scene-relative object motion during self-motion.^40^ We did not find activation in the human visual area V6, located in the parieto-occipital sulcus. V6 is thought to play an important role in the extraction of optic flow for the monitoring and guidance of self-motion (egomotion).^42^

Another hint for visually evoked egomotion comes from the bilateral activation of the CSv. This area is active during visual stimulation provided that visual stimuli are indicative of self-motion.^43,44^ Visual input is provided by V6, the putative intraparietal cortex (pVIP) and the posterior insular cortex. It is active during vestibular stimulation, provides the basis for self-motion and represents a key interface between sensory (vestibular, visual) and motor systems (supplementary pre-motor area, SMA) in the online control of locomotion.^43^ However, the excitability of this core element of human egomotion network^36^ was larger in EA2 patients with higher cerebellar disability scores (SARA) (split-half analysis) but overall did not differ between patients and HP.

This analysis also revealed not only larger activation in CSv but also in V5/MT of patients with higher SARA scores and in patients with symptom onset in late adolescence, as its activation increased with the age at symptom onset of EA2. In turn, early disease onset in EA2 patients was associated with lower MT/V5 excitability in response to visual motion stimulation. In early-onset EA2, these areas may not fully mature, resulting in reduced visual excitability over the life span (age). Patients with later onset may undergo typical age-related development of cortical excitability but its evolution during the life time is counterbalanced due to continuous degeneration of smooth pursuit which is heavily impaired in all older EA2 patients.

Disease manifestation during the maturation of the patients’ brain seems to reduce the natural increase in V5 excitability over the life span as it is reflected by the correlation of V5 excitability to visual motion in our HP with age (Supplementary Fig. 1). This increase in V5 excitability in healthy subjects may counterbalance the age-related decrease in global motion perception (with increasing perception thresholds) as it is associated with age-related morphological changes in the visual cortex, including V5/MT, especially in response to slow visual motion.^45^ This hypothesis would require age-dependent recordings of V5 excitability over the life span in EA2. Age-related decline in BOLD response to checkerboard or optokinetic stimulation has so far not been found in V5/MT of healthy subjects.^46^

Unlike CSv, V5 excitability in EA2 patients was neither correlated with age, nor with the cerebellar disability (SARA score, except for a trend in the split-half analysis) or with disease duration. Additional support for visual–vestibular excitability as a potential trait marker of EA2 comes from the observation that the patients’ lower excitability of the visual cortex is maily observed in unmedicated EA2 patients.

Altered visual sensitivity is typically found in *migraine*, which is also a frequent comorbid disease in EA2 patients. Larger brain excitability in response to sensory stimulation and altered resting-state functional connectivity is found in migraine sufferers with aura.^47^ Bilateral V5 complex was shown to be stronger activated (*hyperexcitable*) by optokinetic visual stimulation in patients with migraine with aura compared with HP.^48^ There were too few EA2 patients in our cohort to contrast those with versus without migraine. Using functional near-infrared spectroscopy, individuals with another disorder with increased visual sensitivity, i.e. visual vertigo, showed lower middle prefrontal activation during OF stimuli possibly affecting the control of the normal reciprocal inhibitory visual-vestibular interaction,^49^ a cornerstone of stable self-motion perception and spatial orientation.

### Visual cortex and motion processing in vestibular disease

As a consequence of vestibulo-cerebellar degeneration EA2 patients suffer from gaze holding deficits, often significant downbeat nystagmus leading to vertical oscillopsia with impaired visual acuity. Physiological strategies to counterbalance oscillopsia are alterations of visual cortex sensitivity, in particular visual motion sensitivity. This functional compensation potentially reduces the annoying aspects of involuntary eye oscillations but alters visual sensitivity for moving targets in the visual surroundings. Visual motion sensitivity has been found to be reduced in patients with cerebellar downbeat nystagmus^22^ and bilateral vestibular hypofunction, in particular during locomotion.^23^

Most of our EA2 patients showed gaze instability with ocular oscillations (commonly downbeat nystagmus) which impair visual acuity not only during locomotion but also under head-stationary conditions. Visual motion perception has not yet been tested in EA2 patients, but it is likely to be reduced as a compensatory mechanism to reduce visual disturbance caused by oscillopsia. Oscillopsia-suppressing mechanisms are well known from patients with congenital (infantile) nystagmus.^50^ Neural activity of motion-sensitive area V5/MT was shown to be reduced in congenital nystagmus and increased when the patient’s nystagmus became smaller during fixating the zero zone, the gaze position in which nystagmus disappears.^51^ Likewise, cerebral blood flow in the visual cortex is deactivated in experimentally induced caloric nystagmus which has been suspected to reduce oscillopsia perception.^52^ Galvanic vestibular stimulation elicits deactivation in primary and secondary visual areas.^53^ Visually induced circular vection strikingly deactivated parieto-insular vestibular cortex areas.^24^ Several lines of evidence support this concept of reciprocal inhibitory visual–vestibular interaction as a multisensory mechanism for self-motion perception as it protects visual self-motion perception from potential vestibular mismatches.^54^ Thus, reduced visual cortex activity in our EA2 patients may reflect the physiological attempt to reduce visual motion perception and oscillopsia related to ocular oscillations (downbeat nystagmus).

### Excitability in the multisensory vestibular cortex

Interestingly, group contrasts revealed stronger activation by visual stimulation in cortical areas of EA2 patients (Fig. 2) involved in vestibular processing, e.g. supramarginal (including Rolandic operculum), retro- and posterior insular cortex adjacent to the IPL as well as superior temporal gyrus.^55-57^ In particular the IPL (PF) is known to be strongly activated during visually induced circular vection eliciting egomotion perception.^58^

The higher responsivity in EA2 patients could be linked to either a larger cortical excitability of these regions receiving visual and vestibular cues related to self-motion or a reduced reciprocal inhibition by the lower activation of the visual cortex. Activation of these multisensory vestibular cortical regions^57^ by visual motion stimulation was not related to the level of cerebellar disability (SARA) or disease duration. Future studies should elucidate whether the increased excitability of the multisensory vestibular cortex regions is related to the abnormal spells of postural imbalance and vestibular perception of EA2 patients.

### Excitability in the cerebellar cortex

There is abundant evidence for visual processing in the cerebellum to guide motor and sensory-motor control of visually guided movements. Interestingly, visual stimulation (OF, CB) elicited lower activation in hemispheric Crus I and II and vermal lobules IV, VII, and IX (Uvula) of the patients. Vermis lobule VII is crucially involved in visually guided movements, particularly smooth pursuit and saccadic eye movements.^59^ The lobules IX and X of the vermis (nodulus and uvula) receive not only vestibular afferents to subserve its role in spatial orientation^60^ but their neurons are also tuned by optic flow.^61^ This remarkable visual–vestibular convergence and the similarity between the nodulus/uvula and the cortical motion-sensitive V5 suggested a functional coupling between the two areas for self-motion processing. Accordingly, the cerebellar uvula has been established as a core part of the human egomotion networks as it is activated during optic flow, particularly when combined visual and vestibular stimulation elicited egomotion perception.^36,58^ Surprisingly, the EA2 patients with medication did not show larger vermis activation compared to the non-treated subgroup. It needs to be investigated in the future whether (i) uvula activation of EA2 patients is lower in response to vestibular and combined visual–vestibular stimulation and (ii) whether it is larger in patients on treatment. This would potentially allow deductions on impaired cerebellar egomotion processing in EA2 patients.

Crus I and II are cerebellar areas engaged in working memory^62^ and internal error prediction for various modalities^63^ as they compare anticipated and actual outcome of a sensory-driven senso-motor command and perception.^64,65^ Psychophysical interaction analyses revealed robust connectivity of the Crus I to the visual motion area V5, strongly modulated by attention.^62^ Along this suggestion, the cerebellum serves as a state (e.g. egomotion) estimator that provides outcome predictions which may also hold for egomotion perceptions. Since our EA2 patients showed lower activity to visual motion stimulation it needs to be investigated whether egomotion perception is impaired in EA2 patients. We recently showed that vestibular egomotion perception thresholds seem to affect postural stability in EA2 patients as postural sway increased with higher egomotion perception thresholds, when proprioceptive information is diminished.^66^

### Modification of the cortical excitability by medication

The key findings of our patients’ activation in the above-mentioned visual–vestibular brain areas strongly differed between patients with and without medication. Although not designed as a therapeutic trial with a prospective placebo-controlled comparative (pre–post) study design the key findings were largely found in non-treated EA2 patients and significantly smaller or even normalized in patients on medication. Our data show that both medications elicit not only beneficial effects on the cerebellar but also extra-cerebellar cortical excitability.

Based on its increase in extracellular proton concentration,^67^ acetazolamide, a carbonic anhydrase inhibitor, inhibits ion permeation through open calcium channels, which are widely distributed in the nervous system and particularly abundant in the cerebellum.^4^ Acetazolamide reduces the number and severity of EA2 vertiginous attacks with postural imbalance^68, 69, 70^ presumably by stabilizing the ion channels which are dysfunctional in *CACNA1A* mutations. It has been shown to be equally effective in reducing the number of attacks in EA2 compared to fampridine, the prolonged-release form of 4-AP.^68^ The potassium-channel blocker, 4-AP not only improves axonal conductance but also neuronal excitability, particularly in the cerebellum. Alterations in the intrinsic properties of Purkinje neurons with irregular firing and synaptic dysfunction caused by mutations in the P/Q type voltage-gated calcium channels has been suspected to cause EA2 attacks.^4,71^ This loss in the precision of Purkinje cell pacemaking is a consequence of mutant calcium channels and was modified by changing activity of small-conductance calcium-dependent potassium channels.^72^ Thereby, the potassium-channel blocker, 4-AP, and its precursors help to increase and synchronize the excitability of Purkinje cells, with beneficial effects on various oculomotor^73,74^ and postural^75^ functions in patients with cerebellar diseases, including EA2 patients tested in double-blinded trials^68,76^ and the recently characterized SCA 27B.^77^

We here provide new lines of evidence for an extra-cerebellar, i.e. supratentorial cortical mode of action for both acetazolamide and 4-AP. Patients with medication showed smaller abnormal excitability of the visual cortex which could be secondary to the reduction of gaze-holding deficits, particularly downbeat nystagmus, usually found in episodic ataxia (unpublished observation). Using the concept of reciprocal inhibitory visual–vestibular interaction smaller activity would be expected in the vestibular processing brain areas, e.g. the posterior insula and IPL. However, we found the opposite, an increase in activity suggesting that medication in EA2 patients increases the excitability of multisensory vestibular cortical areas or it weakens reciprocal inhibitory visual–vestib+ular interactions. This needs to be tested with combined visuo-vestibular stimulation in EA2 patients.

## Limitations

Neural activity in response to visual stimuli was not recorded during a circumscribed episode of visual blurring and postural unsteadiness. Our conclusions are constrained to the interval between EA2 attacks. Moreover, abnormal excitability may be masked by medications (acetazolamide and fampridine) but except for one patient none of the patients were free of attacks. As we did not intend to make a pre–post study design, the therapeutic conclusions are restricted but promising. Furthermore, a double-blinded, crossover medication study design would be highly interesting, but it would raise ethical concerns, as patients could experience an increase in attacks during the washout phase without medication. Given the rarity of the disease, patient recruitment was challenging. As a result, the number of EA2 patients included in the study was limited, and the small sample size—particularly in subgroup analyses—should be taken into consideration when interpreting the results.

## Conclusion

EA2 patients show lower brain excitability by visual and visual motion stimuli in primary visual cortex areas but larger activation in multisensory vestibular cortical regions. This might either reflect abnormal visual–vestibular interaction or altered excitability in vestibular and visual cortex areas, possibly facilitating the attacks with postural imbalance. Either explanation should be tested by comparing brain activity in these regions during combined visual-vestibular stimulation.

## Supplementary Material

fcaf400_Supplementary_Data

## Data Availability

The data that support the findings of this study are available from the corresponding author, upon reasonable request.
