# Supplementary material for: Dissociable effects of medication on visual–vestibular brain excitability by visual motion stimuli in episodic ataxia type 2
Source: Brain Commun. 2025 Oct 24;7(5):fcaf400. doi: 10.1093/braincomms/fcaf400 (PMC12569760; doi:10.1093/braincomms/fcaf400)
Supplement: fcaf400_Supplementary_Data [file fcaf400_supplementary_data.docx]

# **Supplementary material**

## **Supplementary Results**

*Correlation of clinical data with ROI activity*

Additionally, the age of HP correlated with results in the area V5 bilaterally (left r = 0.57, p = 0.008, right r = 0.47, p = 0.035) during OF (versus rest) and in the right V5 (r = 0.45, p = 0.044) and in the left PF (r = 0.54, p = 0.013) during CB (versus rest). See also Supplementary Figure 1.

## **Supplementary Figure**


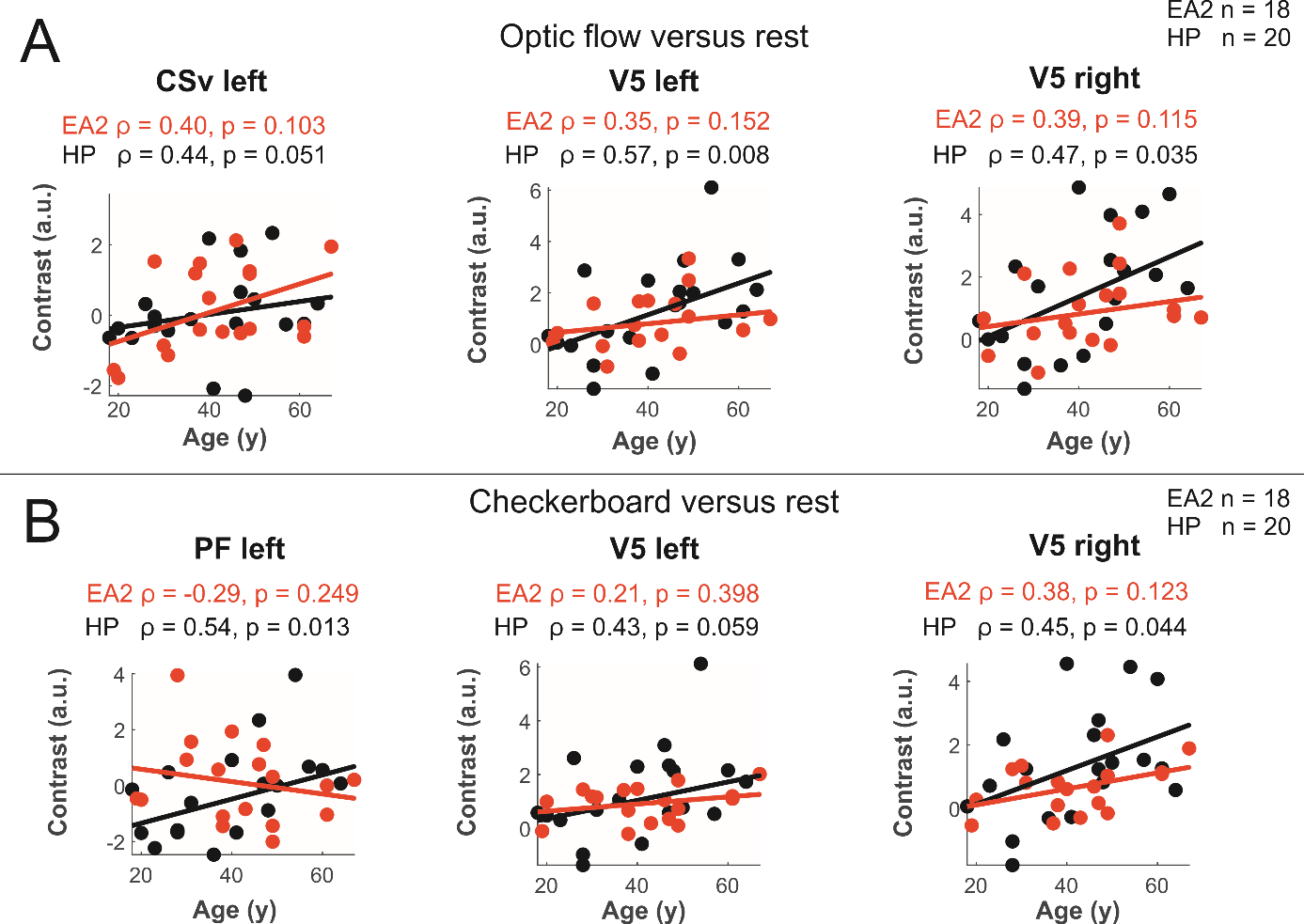


**Supplementary Figure 1. Correlation Between Age and Activation in Left CSv, left PF and Bilateral V5 in Healthy Participants and EA2 Patients during optic flow or checkerboard stimulation.**

In healthy participants (HP, black), activation in the left CSv and bilateral V5 regions show a significant correlation with age during Optic Flow (versus Rest). Additionally HP show significant correlation between age and activation in the left PF and in bilateral V5 regions. Conversely, EA2 patients (EA2, red) display no such correlation between activation in these regions and age. A Spearman’s rho correlation coefficient was used for this analysis. The corresponding ρ- and p-values are provided above the plots. N = Number of participants in this analysis.

## **Supplementary Tables**

**Supplementary Table 1**

| **Main effect group: EA2 patients > healthy controls** | | | | | |
| --- | --- | --- | --- | --- | --- |
| Brain area | side | Cytoarchitectonic area | cluster size | *t*-value | *x y z* (mm) |
| Sub-Gyral | L | CA1 (PhG, CA2) | 45 | 14.38 | -27 -44 0 |
| SupraMarginal | R | PFcm (PF) | 209 | 12.80 | 58 -24 28 |
| SupraMarginal R | R |  |  | 9.81 | 63 -32 40 |
| Rolandic Oper R | R |  |  | 8.36 | 50 -22 18 |
| Temporal Sup | L | Area Ia1 (Id3, Ia3) | 53 | 10.18 | -40 -4 -10 |
| Sub-Gyral | R | Area 6d3 | 36 | 9.73 | 23 -2 45 |
| Frontal Sup | R |  |  | 7.24 | 20 8 55 |
| Temporal Sup | R | Area STS1 (STS2, Te3) | 20 | 9.59 | 56 -12 -8 |
| Temporal Sup | R |  |  | 6.61 | 63 -7 -8 |
| Hippocampus | R | CA3 (CA2, DG, Subc.ProS, Subc.Sub) | 11 | 9.49 | 30 -20 -10 |
| Rolandic Oper | R | Area 6r1 (Op4, 6v3) | 45 | 8.95 | 53 6 15 |
| Frontal Mid | L | Area MFG4 (SEG4) | 100 | 8.93 | -30 33 22 |
| Frontal Inf Tri | L |  |  | 7.13 | -42 33 22 |
| Sub-Gyral | L |  |  | 6.69 | -27 20 28 |
| Parietal Inf | R | Area PFm (PF, hIP2) | 25 | 8.91 | 56 -40 50 |
| Cerebellum Lobule VI | R |  | 68 | 8.81 | 18 -62 -15 |
| Cerebellum Lobule VI | R |  |  | 8.09 | 26 -54 -20 |
| Temporal Sup | L | PFcm (TPJ, PFop) | 69 | 8.55 | -57 -32 20 |
| Temporal Sup | L |  |  | 6.40 | -44 -32 18 |
| Rolandic Oper | L |  |  | 6.39 | -40 -24 20 |
| Parietal Inf | L | PFt (Area2, 3b, PFop) | 64 | 8.36 | -50 -24 35 |
| Parietal Inf | L |  |  | 7.02 | -47 -32 40 |
| Cingulate Gyrus | R |  | 12 | 8.35 | 18 -10 28 |
| Temporal Sup | L | Area Te 2.1 (STS1, Te3, Te1) | 15 | 8.33 | -54 -14 2 |
| Temporal Sup | R | Temporal-to-parietal (STS1) | 20 | 8.30 | 48 -37 5 |
| Thalamus | R | CL (MD, PUa) | 27 | 7.99 | 8 -27 8 |
| Temporal Pole Sup | R | Area TI (Temporal-to-parietal, Tel, STS2) | 49 | 7.77 | 48 13 -18 |
| Insula | R |  |  | 7.60 | 50 8 -5 |
| Temporal Pole Sup | R |  |  | 6.60 | 48 6 -12 |
| Temporal Mid | L | Temporal-to-parietal | 19 | 7.71 | -62 -54 -2 |
| Temporal Mid | L |  |  | 6.15 | -57 -64 2 |
| Sub-Gyral | R | Area hIP8 (hIP5, hIP7) | 12 | 7.26 | 30 -62 25 |
| Occipital Mid | R |  |  | 6.10 | 33 -64 35 |
| Cingulum Ant | R | Frontal-to-occipital | 21 | 7.10 | 3 30 25 |
| Postcentral | L | Area Op1 | 11 | 6.78 | -60 -20 18 |
| **Main effect group: controls > patients** | | | | | |
| Brain area | side | Cytoachitectonic area | cluster size | *t*-value | *x y z* (mm) |
| Occipital Sup | R | hOc4 (hPO1, hIP7, hOc3d) | 2686 | 19.07 | 20 -82 30 |
| Angular | R |  |  | 14.31 | 53 -57 30 |
| Calcarine | R |  |  | 14.24 | 10 -94 0 |
| Angular | L | PFm, (PGa, Temporal-to-Parietal; PGP, PF) | 1029 | 16.03 | -47 -57 30 |
| Temporal Mid | L |  |  | 13.57 | -50 -54 15 |
| Angular | L |  |  | 13.05 | -50 -64 38 |
| Supp Motor Area | R | Area 6d2 (8d1) | 1387 | 15.21 | 13 16 58 |
| Frontal Sup Medial | R |  |  | 10.23 | 10 40 48 |
| Frontal Sup | R |  |  | 9.31 | 18 43 42 |
| Paracentral Lobule | L | Area 6d1 (Area 4a) | 61 | 14.42 | -10 -20 72 |
| Medial Frontal Gyrus | L |  |  | 6.31 | -14 -22 60 |
| Temporal Mid | L | Area STS2 (STS1) | 142 | 14.22 | -52 -4 -20 |
| Temporal Mid | L |  |  | 12.14 | -54 6 -22 |
| Frontal Sup | L | Area SFG2 (SFG4) | 204 | 13.41 | -12 46 42 |
| Frontal Sup | L |  |  | 10.81 | -20 50 35 |
| Frontal Sup Med. | L |  |  | 10.24 | -7 58 30 |
| Temporal Mid | R | Area STS2 (STS1, Temporal-to-parietal) | 159 | 12.51 | 58 -7 -18 |
| Temporal Mid | R |  |  | 10.22 | 56 -3-22 |
| Temporal Mid | R |  |  | 8.48 | 48 0 -25 |
| Supp Motor Area | L | Area 6ma (6d2) | 87 | 12.27 | -4 16 60 |
| Supp Motor Area | L |  |  | 8.21 | -7 8 65 |
| Frontal Sup | L |  |  | 7.70 | -12 26 55 |
| Cerebellum Crus I | L |  | 70 | 11.91 | -30 -82 -22 |
| Occipital Inf | L |  |  | 8.76 | -34 -80 -10 |
| Sub-Gyral | L | Area 3b (5L, 2, 5Ci) | 142 | 11.76 | -17 -37 58 |
| Postcentral | L |  |  | 9.87 | -24 -34 68 |
| Precuneus | L |  |  | 9.11 | -12 -40 68 |
| Frontal Mid | L | Area 8v1 (Frontal-II, 44) | 258 | 11.57 | -40 16 50 |
| Sub-Gyral | L |  |  | 10.06 | -20 -7 42 |
| Frontal Inf Oper | L |  |  | 10.05 | -40 13 32 |
| Frontal Inf Tri | L | Area 44 (45, Op9) | 154 | 11.21 | -54 23 8 |
| Frontal Inf Oper | L |  |  | 8.00 | -47 16 15 |
| Frontal Inf Tri | L |  |  | 7.29 | -40 18 18 |
| Fusiform | R | Temporal-to-Parietal (Subc.ProS, Subc.Sub) | 75 | 11.14 | 26 -30 -18 |
| Fusiform | R |  |  | 8.82 | 33 -40 -20 |
| Postcentral | R | Area 3b (5L,, 2, 5M, 4a) | 262 | 10.37 | 16 -37 62 |
| Sub-Gyral | R |  |  | 10.26 | 18 -22 58 |
| Precentral | R |  |  | 10.16 | 20 -17 68 |
| Cerebellum Crus II | R |  | 277 | 10.25 | 30 -80 -38 |
| Cerebellum Crus II | R |  |  | 9.62 | 16 -84 -38 |
| Fastigium | L |  |  | 8.31 | -7 -60 -30 |
| Cingulate Gyrus | R |  | 97 | 9.73 | 18 16 40 |
| Frontal Sup | R |  |  | 9.46 | 26 28 50 |
| Frontal Mid | R |  |  | 6.22 | 30 20 52 |
| Temporal Inf | L |  | 33 | 9.67 | -62 -27 -18 |
| Temporal Mid | L |  |  | 8.97 | -54 -30 -15 |
| Cerebellum IX | R | Dorsal Dentate Nucleus (Ventral Dentate Nucleus) | 12 | 9.31 | 13 -47 -38 |
| Cerebellum Crus II | L |  | 60 | 9.08 | -20 -82 -35 |
| Cerebellum Crus II | L |  |  | 6.33 | -24 -80 -45 |
| Cerebellum Crus I | L |  |  | 8.96 | -12 -84 -20 |
| Cerebellum Lobule VI | L |  |  | 8.25 | -20 -74 -18 |
| Sub-Gyral | L | Area ATS1 | 38 | 8.78 | -44 -30 -5 |
| Temporal Mid | L |  |  | 7.04 | -60 -14 -5 |
| Temporal Mid | L |  |  | 7.03 | -52 -22 -5 |
| Frontal Mid | R | Frontal-II (Area 6v2, IFS3) | 36 | 8.48 | 46 16 42 |
| Frontal Mid | R |  |  | 7.98 | 40 23 42 |
| Frontal Inf Orb | R |  |  | 8.22 | 46 28 -10 |
| Fusiform | L | Temporal-to-parietal (CA1m FG3) | 44 | 7.73 | -30 -32 -20 |
| Parahippocampal | L |  |  | 7.48 | -22 -27 -12 |
| Parahippocampal | L |  |  | 6.92 | -24 -24 20 |
| Frontal Sup Medial | L | Area p32 (Fp2, SFG3, Fp1) | 16 | 7.44 | -10 58 15 |
| Frontal Sup | R | Area (FG2, SFG2, Fp1, SFS1) | 25 | 7.21 | 18 60 20 |
| Frontal Sup Medial | R |  |  | 6.75 | 8 58 10 |
| Parietal Sup | R | Area 7A (5l, 7PC, hIP3) | 36 | 6.98 | 23 -57 65 |
| Parietal Sup | R |  |  | 6.52 | 28 -52 55 |

**Supplementary Table 2**

| **Main effect of condition Optic flow (versus rest):** | | | | | | | |
| --- | --- | --- | --- | --- | --- | --- | --- |
| Brain area | side | Cytoarchitectonical area | | cluster size | | *t*-value | *x y z* (mm) |
| Calcarine | R | hOc1 (hOc2) | | 6363 | | 48.59 | 6 -82 0 |
| Occipital Sup | L | hOc2 (hOc1, 3d) | |  | | 40.51 | -10 -100 10 |
| Calcarine | R | hOc1 (hOc2) | |  | | 40.15 | 10 -94 2 |
| Thalamus | L | PUI | | 20 | | 13.56 | -20 -30 -2 |
| **Main effect of Checkerboard (versus rest):** | | | | | | | |
| Brain area | side | **Cytoarchitectonical area** | | cluster size | | *t*-value | x y z (mm) |
| Calcarine | R | hOc1 (hOc2) | | 8068 | | 39.16 | 6 -82 0 |
| Lingual | R | hOc1 (hOc2, 3v) | |  | | 33.87 | 10 -70 2 |
| Calcarine | R | hOc1 (hOc2, 3d, 4d) | |  | | 32.15 | 13 -87 12 |
| Thalamus | L | PUI | | 39 | | 13.29 | -20 -30 -2 |
| Hippocampus | R | PUI, CGL, Subc.Sub, Subc.ProS | | 17 | | 11.61 | 23 -27 -5 |
| Cerebellum VIII | L | Dorsal dentate Nucleus | | 218 | | 9.96 | -24 -67 -45 |
| Cerebellum VIII | L | Dorsal dentate Nucleus | |  | | 8.20 | -30 -60 -42 |
| **Main effect of Optic flow > Ceckerboard:** | | | | | | | |
| Brain area | side | Cytoarchitectonical area | | cluster size | | *t*-value | x y z (mm) |
| Occipital Mid | L | hOc1 (hOc2, 3d) | | 145 | | 9.78 | -12 -100 5 |
| Lingual | L | hOc3 (hOc2, 4) | |  | | 8.21 | -14 -87 -10 |
| Cuneus | R | hOc1 (hOc2, 3d, 3v) | | 221 | | 9.21 | 18 -94 10 |
| Lingual | R | hOc2 (hOc3v, 1, 4v) | |  | | 8.96 | 16 -87 -8 |
| Cuneus | R | hOc1 (hOc2) | |  | | 8.76 | 10 -97 5 |
| **Main effect of Checkerboard > Optic flow:** | | | | | | | |
| Brain area | side | | Cytoarchitectonical area | cluster size | *t*-value | x y z (mm) | |
| Calcarine | L | | hOc1 (hoC6, 2, 3d, 3v) | 501 | 9.89 | -10 -74 10 | |
| Lingual | L | | hOc1 (3, 3v) |  | 9.18 | -14 -62 2 | |
| Calcarine | R | | hOc1 (2, 3d, 6) |  | 8.45 | 13 -70 12 | |

**Supplementary Table 3**

| **EA2 patients with medication > healthy participants** | | | | | |
| --- | --- | --- | --- | --- | --- |
| Brain area | side | Cytoarchitectonical area | cluster size | *t*-value | *x y z* (mm) |
| SupraMarginal | R | AreaPFcm (Area TPJ, IPL) | 1918 | 21.45 | 58 -27 32 |
| Rolandic Oper | R |  |  | 14.41 | 56 6 12 |
| SupraMarginal | R |  |  | 14.06 | 63 -20 18 |
| Supp Motor Area | L | Area 6d1 (PreCG,, Area 6d2) | 833 | 14.59 | -10 -10 70 |
| Sub-Gyral | R |  |  | 12.75 | 23 0 45 |
| Supp Motor Area | R |  |  | 11.65 | 8 6 65 |
| Temporal Sup | L | Area PFcm (Area TPJ, PFop) | 771 | 14.48 | -57 -32 20 |
| SupraMarginal | L |  |  | 11.86 | -50 -27 32 |
| Precentral | L |  |  | 11.76 | -52 3 18 |
| Lingual Gyrus | L | hOc1 (hOc4v, hOc3v) | 24 | 14.36 | -20 -74 0 |
| Cerebelum 4 5 | L |  | 20 | 13.55 | -10 -40 -12 |
| Heschl | L | Area Id1 (Insula, Area Id3) | 135 | 13.54 | -40 -17 10 |
| Insula | L |  |  | 9.90 | -40 -7 0 |
| Temporal Sup | L |  |  | 8.96 | -40 -4 -10 |
| Frontal Mid | L | Area MFG4 (Area SFG4) | 331 | 12.75 | -30 33 25 |
| Frontal Inf Tri | L |  |  | 10.09 | -42 30 25 |
| Sub-Gyral | L |  |  | 9.99 | -30 26 20 |
| Lingual | R | Frontal-to-temporal | 51 | 12.48 | 16 -30 -10 |
| Cerebellum 4 5 | R |  |  | 9.24 | 10 -40 -12 |
| ParaHippocampal | R |  |  | 6.79 | 23 -34 -8 |
| Cerebellum 6 | R | Area FG1 (FUsG, FG2-4) | 90 | 12.06 | 23 -60 -18 |
| Fusiform | R |  |  | 8.69 | 36 -57 -20 |
| Precuneus | L | Area 5L (SPL, Area 7A, 5M) | 59 | 11.85 | -10 -47 68 |
| Paracentral Lobule | L |  |  | 9.97 | -4 -32 65 |
| Sub-Gyral | R | FuP (Ventral Striatum, Fundus of Putamen) | 57 | 11.41 | 18 23 -12 |
| Frontal Mid Orb | R |  |  | 7.72 | 26 33 -18 |
| Insula | R |  |  | 6.38 | 30 20 -8 |
| Cerebellum 6 | L |  | 68 | 11.35 | -20 -62 -18 |
| Cerebellum 6 | L |  |  | 8.51 | -30 -62 -22 |
| Sub-Gyral | L | CA1 (Hippocampus, DG, CA2) | 30 | 11.15 | -24 -42 0 |
| Frontal Mid | R | Area IFS1 (MFG4) | 431 | 10.85 | 33 30 32 |
| Frontal Sup | R |  |  | 10.80 | 26 63 15 |
| Frontal Sup | R |  |  | 10.57 | 23 56 2 |
| Precentral | L | Area 6d1 (PreCG, Area 6d3) | 27 | 10.67 | -30 -14 58 |
| Precentral | L |  |  | 7.44 | -32 -17 65 |
| Fusiform | L | Area FG3 (FusG, Ph2) | 22 | 10.67 | -27 -40 -22 |
| Sub-Gyral | L | FuCd (ventral Striatum, Fundus of Caudate) | 37 | 10.47 | -17 23 -10 |
| Frontal Mid Orb | L |  |  | 10.15 | -17 33 -15 |
| Frontal Sup | L | Area 6d3 (6d2) | 56 | 9.99 | -17 8 55 |
| Frontal Sup | L |  |  | 8.33 | -20 3 65 |
| Cuneus | R | Frontal-to-Occipital | 177 | 9.96 | 16 -80 45 |
| Sub-Gyral | R |  |  | 9.62 | 30 -62 25 |
| Occipital Mid | R |  |  | 9.07 | 30 -74 38 |
| Temporal Inf | L | Area FG4 (FusG) | 11 | 9.62 | -47 -52 -8 |
| Fusiform | L | Area FG4 (FusG) | 10 | 9.43 | -42 -47 -20 |
| Insula | L | Area Id7 | 64 | 8.84 | -32 23 2 |
| Insula | L | Area Id6 |  | 7.32 | -40 13 0 |
| Precentral | L | Area 3b, Area 1 | 34 | 8.70 | -40 -17 50 |
| Cuneus | L | Area hOc1 | 28 | 8.40 | -7 -80 15 |
| Calcarine | L | Area hOc1 |  | 8.25 | -12 -87 12 |
| Cuneus | L | Area hOc2 |  | 6.41 | -4 -90 15 |
| Thalamus | R | PUm, LP | 10 | 7.91 | 13 -32 12 |
| Temporal Mid | L | Temporal-to-Parietal | 30 | 7.87 | -54 -57 2 |
| Temporal Mid | L |  |  | 7.07 | -47 -54 5 |
| Temporal Mid | L |  |  | 6.92 | -54 -67 0 |
| Parietal Inf | R | Area hIP6 | 12 | 7.76 | 43 -57 45 |
| Paracentral Lobule | R | Area 5M | 18 | 7.69 | 8 -34 52 |
| Lingual | L | AreahOc2 | 10 | 7.62 | -7 -77 0 |
| Precuneus | L | Frontal-to-occipital | 15 | 7.32 | -10 -74 38 |
| Superior Temporal Gyrus | R | Area TI | 18 | 6.96 | 43 -17 -5 |
| Sub-Gyral | R |  |  | 6.90 | 40 -10 -10 |
| **Healthy participants > EA2 patients with medication** | | | | | |
| Brain area | side | Cytoarchitectonical area | cluster size | *t*-value | *x y z* (mm) |
| Occipital Mid | L | Area hOc1 | 1657 | 20.52 | -12 -100 2 |
| Occipital Sup | R | Area hOc2 |  | 18.13 | 16 -94 20 |
| Calcarine | R | Area hOc1 |  | 15.05 | 8 -92 2 |
| Temporal Mid | L | Area STS2 | 308 | 17.19 | -54 -2 -20 |
| Temporal Mid | L |  |  | 11.44 | -52 -12 -8 |
| Temporal Mid | L |  |  | 11.42 | -62 -10 -18 |
| Superior Temporal Gyrus | L | Area hIP6 | 590 | 14.94 | -34 -57 28 |
| Temporal Mid | L | Temporal-to-Parietal |  | 14.04 | -50 -54 18 |
| Superior Temporal Gyrus | L |  |  | 13.03 | -42 -50 18 |
| ParaHippocampal | R | CA1 | 64 | 14.80 | 30 -30 -15 |
| Hippocampus | R | CA1 | -1 | 10.03 | 38 -20 -15 |
| Cerebellum Crus1 | L | Area hOc4v | 158 | 14.03 | -32 -80 -20 |
| Cerebellum Crus1 | u |  |  | 9.45 | -10 -84 -18 |
| Lingual | L |  |  | 9.29 | -20 -80 -15 |
| Supp Motor Area | L | Area 6ma | 34 | 12.57 | -7 16 60 |
| Temporal Pole Mid | R | Temporal-to-Parietal | 180 | 12.25 | 46 10 -35 |
| Temporal Pole Mid | R |  |  | 11.19 | 56 6 -22 |
| Temporal Mid | R |  |  | 11.01 | 58 -10 -18 |
| Frontal Inf Oper | L | Area IFJ1 | 23 | 12.04 | -40 13 32 |
| Cerebellum 9 | R | Ndentd (dorsal dentate nucleus) | 73 | 11.89 | 10 -52 -42 |
| Cerebellum 8 | R |  |  | 7.04 | 20 -62 -40 |
| Cerebelum Crus1 | L |  | 39 | 11.53 | -20 -82 -32 |
| Frontal Inf Tri | L | Area 45 (IFG) | 73 | 11.42 | -54 26 10 |
| Frontal Inf Tri | L |  |  | 9.32 | -47 26 2 |
| Medial Frontal Gyrus | R | Area 4a (PreCG) | 36 | 11.13 | 13 -22 60 |
| Paracentral Lobule | R |  |  | 8.97 | 8 -24 70 |
| Sub-Gyral | R |  |  | 8.23 | 20 -24 50 |
| Cerebellum Crus2 | R |  | 111 | 10.90 | 30 -80 -35 |
| Cerebellum Crus2 | R |  |  | 9.43 | 13 -84 -32 |
| Paracentral obule | L | Area 4a (PreCG) | 22 | 10.29 | -10 -22 70 |
| Cingulate Gyrus | R |  | 28 | 9.84 | 18 16 40 |
| Sub-Gyral | R |  |  | 7.62 | 20 20 32 |
| Fastigium | L | Ninterp (interposed nucleus) | 198 | 9.81 | -7 -60 -30 |
| Cerebellum 8 | R | Ndentd (dorsal dentate nucleus) |  | 9.46 | 8 -70 -32 |
| Cerebellar Tonsil | L |  |  | 9.28 | -22 -40 -40 |
| Frontal Inf Orb | L | Frontal-to-Temporal | 20 | 9.50 | -44 30 -12 |
| Temporal Pole Sup | R | Temporal-to-parietal | 12 | 9.50 | 33 13 -28 |
| Sub-Gyral | L |  | 26 | 9.41 | -30 -7 38 |
| Precentral Gyrus | L |  |  | 9.35 | -37 -4 30 |
| Sub-Gyral | L |  |  | 8.14 | -20 -7 42 |
| ParaHippocampal | L | Subc.ProS (Hippocampus) | 37 | 9.27 | -27 -22 -20 |
| Fusiform | L |  |  | 8.23 | -34 -27 -22 |
| Parahippocampal | L |  |  | 7.75 | -24 -30 -10 |
| Sub-Gyral | R | Area Ph2 (PhG) | 16 | 9.26 | 38 -37 -10 |
| Fusiform | R |  |  | 8.89 | 33 -40 -18 |
| Temporal Pole Sup | L | Area PirT.Tit (PiriformCortexMesial) | 15 | 9.15 | -30 8 -25 |
| Sub-Gyral | L | Area 3b | 22 | 8.99 | -17 -37 58 |
| Precuneus | L |  |  | 8.34 | -12 -40 68 |
| Vermis 4 5 | L |  | 10 | 8.82 | -2 -47 -8 |
| Lentiform Nucleus | L | Area Ch4 | 12 | 8.68 | -22 -10 -5 |
| Culmen | L | Area 5L | 11 | 8.47 | -10 -44 -25 |
| Frontal Sup | L | Area SFG2 | 20 | 8.26 | -12 46 42 |
| Temporal Mid | L | Area STS1 | 20 | 8.03 | -47 -30 -2 |
| Cerebellum 8 | L | Ndentd (dorsale dentate nucleus) | 18 | 7.78 | -24 -60 -45 |
| Pyramis | L |  |  | 7.58 | -22 -62 -38 |
| Frontal Med Orb | R | Area Fp2 | 18 | 7.75 | 8 56 -8 |
| Frontal Sup Medial | R |  |  | 7.37 | 6 63 2 |
| Rectus | L | Area Fp2 | 12 | 7.66 | -2 48 -15 |
| Frontal Sup | R | Area SFG4 | 11 | 7.27 | 20 43 38 |
| Occipital Mid | L | Area hOc4lp | 13 | 7.16 | -30 -87 20 |
| **EA2 patients without medication > healthy participants** | | | | | |
| Brain area | side | Cytoarchitectonical area | cluster size | *t*-value | *x y z* (mm) |
| Lateral Ventricle | R | Frontal-to-Temporal-II | 36 | 22.70 | 28 -17 -10 |
| Sub-Gyral | L | CA1 (Hippocampus) | 48 | 19.46 | -27 -44 0 |
| Hippocampus | L |  |  | 7.83 | -20 -37 -2 |
| Temporal Sup | R | Area STS1 | 60 | 17.83 | 56 -12 -8 |
| Temporal Sup | R |  |  | 7.38 | 46 -10 -12 |
| Temporal Pole Sup | R | Temporal-to-parietal | 17 | 14.21 | 48 13 -20 |
| Hippocampus | L | Frontal-to-Temporal-II | 29 | 11.43 | -34 -24 -10 |
| Hippocampus | L |  |  | 9.03 | -32 -12 -15 |
| Sub-Gyral | R | Temporal-to-Parietal | 11 | 11.31 | 40 -7 -28 |
| Insula | L | Area Ia1 (Insula) | 80 | 11.16 | -37 -4 -10 |
| Temporal Sup | L |  |  | 10.03 | -52 3 -12 |
| Temporal Sup | L |  |  | 9.52 | -54 -7 -8 |
| Hippocampus | R | CA1 | 13 | 9.84 | 33 -37 -2 |
| Cerebellum 6 | R |  | 29 | 9.68 | 26 -52 -22 |
| Cerebellum 8 | R | Ndentd (dorsale dentate nucleus) | 10 | 9.59 | 16 -52 -42 |
| Hippocampus | L | CA2 | 15 | 9.57 | -20 -40 8 |
| Thalamus | R | MD (Thalamus, mediodorsal nucleus) | 13 | 9.22 | 6 -27 8 |
| Cerebellum 8 | L | Ndentd (dorsale dentate nucleus) | 20 | 8.86 | -14 -60 -38 |
| Thalamus | R | VA (Thalamus, ventral anterior nucleus) | 14 | 8.77 | 6 -7 -2 |
| Occipital Mid | L | Area hOc5 | 11 | 7.88 | -40 -77 2 |
| **Healthy participants > EA2 patients without medication** | | | | | |
| Brain area | side | Cytoarchitectonical area | cluster size | *t*-value | *x y z* (mm) |
| Frontal Sup Medial | L | Area SFG2 | 4912 | 23.09 | -10 46 42 |
| Paracentral Lobule | L |  |  | 22.74 | -12 -20 72 |
| Supp Motor Area | R |  |  | 22.55 | 13 18 58 |
| Lingual | R | Area hOc1 | 5707 | 23.08 | 13 -70 2 |
| Occipital Sup | R |  |  | 23.05 | 20 -80 30 |
| Angular | R |  |  | 19.97 | 53 -57 30 |
| Heschl | R | Area Te1.0 | 338 | 20.20 | 50 -12 5 |
| Rolandic Oper | R |  |  | 11.01 | 50 -10 15 |
| Insula | R |  |  | 10.30 | 38 -14 15 |
| Temporal Mid | R | Temporal-to-parietal | 213 | 17.34 | 60 -14 -20 |
| Temporal Mid | R |  |  | 16.88 | 58 -7 -18 |
| Temporal Inf | R |  |  | 9.79 | 53 -17 -28 |
| Postcentral | L | Area 3b | 408 | 16.29 | -22 -34 68 |
| Postcentral | L |  |  | 14.03 | -27 -44 60 |
| Sub-Gyral | L |  |  | 13.66 | -17 -37 58 |
| Temporal Mid | L | Area STS2 | 87 | 15.95 | -54 6 -25 |
| Temporal Mid | L |  |  | 15.83 | -52 -7 -20 |
| Temporal Inf | L |  |  | 7.77 | -50 -17 -28 |
| Temporal Inf | L | Temporal-to-parietal | 91 | 15.90 | -62 -27 -18 |
| Temporal Mid | L |  |  | 15.00 | -54 -32 -12 |
| Temporal Mid | L |  |  | 8.06 | -62 -40 -8 |
| Cerebellum 4 5 | R |  | 133 | 12.64 | 18 -40 -18 |
| Fusiform | R |  |  | 12.04 | 30 -50 -15 |
| ParaHippocampal | R |  |  | 11.57 | 26 -27 -15 |
| Extra-Nuclear | R |  | 50 | 12.61 | 0 -14 -5 |
| Frontal Inf Orb | R | Frontal-to-Temporal I | 80 | 12.18 | 46 28 -10 |
| Frontal Inf Orb | R |  |  | 7.83 | 40 38 -8 |
| Frontal Inf Tri | R |  |  | 7.76 | 48 33 2 |
| Cerebellum 4 5 | L |  | 83 | 12.09 | -20 -37 -28 |
| Temporal Inf | L |  |  | 10.39 | -44 -24 -20 |
| Fusiform | L |  |  | 9.81 | -32 -32 -15 |
| Cingulum Ant | L | Frontal-to-occipital | 17 | 10.61 | -2 0 30 |
| Extra-Nuclear | L | Area p24an.p24a (pACC) | 24 | 10.58 | -2 28 12 |
| Extra-Nuclear | R |  |  | 6.82 | 8 30 10 |
| Parietal Sup | L | Area 7A (SPL) | 44 | 10.39 | -22 -62 60 |
| Parietal Sup | L |  |  | 7.31 | -27 -67 55 |
| Temporal Sup | R | Area PGa (IPL) | 56 | 10.10 | 63 -44 18 |
| Temporal Mid | R |  |  | 8.50 | 53 -42 10 |
| Temporal Mid | R |  |  | 7.17 | 60 -40 8 |
| Frontal Sup | R | Area p32 (pACC) | 20 | 9.90 | 18 50 0 |
| Sub-Gyral | R |  |  | 6.68 | 23 43 5 |
| Parietal Inf | L | Area PFm (IPL) | 25 | 9.57 | -47 -50 52 |
| Parietal Inf | L |  |  | 6.76 | -50 -52 45 |
| Precuneus | L | Area 7A (SPL= | 69 | 9.32 | -2 -60 55 |
| Precuneus | R |  |  | 8.80 | 3 -54 48 |
| Precuneus | L |  |  | 7.97 | -10 -52 42 |
| Thalamus | R | CL (Thalmus, anterior intralaminar nuclei) | 37 | 9.26 | 10 -12 10 |
| Precentral | R | Area 6r1 | 10 | 9.22 | 60 3 18 |
| Cingulum Ant | R | Area p24c.pv24c (pACC) | 13 | 9.20 | 8 36 -2 |
| Vermis 6 | R |  | 32 | 9.13 | 6 -70 -22 |
| Vermis 6 | L |  |  | 6.53 | 0 -60 -20 |
| Cerebellum Crus2 | L |  | 34 | 8.94 | -22 -82 -38 |
| Cerebellum Crus2 | L |  |  | 6.60 | -30 -80 -40 |
| Insula | R | Area Op4 (POperc) | 15 | 8.82 | 46 -2 2 |
| Caudate | L |  | 22 | 8.23 | -12 13 12 |
| Caudate | L |  |  | 7.06 | -14 6 20 |
| Precentral | L | Area 3b (PostCG) | 12 | 8.17 | -52 -4 28 |
| Caudate | R |  | 14 | 7.76 | 16 6 18 |

**Supplementary Table 4**

|  | **SARA ≥ 4** | **SARA < 4** | **Level of significance** |
| --- | --- | --- | --- |
| **Number of patients** | 8 | 10 |  |
| **age (yrs)** | 47.0 ± 9.6 | 37.7 ± 15.1 | n.s. |
| **sex (n female / male)** | 3 / 5 | 4 / 6 | n.s. |
| **age at diagnosis** | 12.5 ± 7.0 | 9.2 ± 4.8 | n.s. |
| **disease duration (yrs)** | 34.5 ± 13.6 | 25.9 ± 14.3 | n.s. |
| **attacks / month** | 6.0 ± 7.9 | 3.75 ± 5.9 | n.s. |
| **attack duration (hrs)** | 4.5 ± 4.7 | 12.36 ± 30.5 | n.s. |
| **SARA** | 6.94 ± 3.11 | 1.70 ± 1.27 | 0.000 |
| **INAS** | 6.37 ± 3.54 | 5.1 ± 2.73 | n.s. |
| **INAS, occular part** | 3.5 ± 2.56 | 3.5 ± 2.32 | n.s. |
| **DHI** | 54.00 ± 26.40 | 26.80 ± 21.02 | 0.027 |
| **CVS** | 6.75 ± 4.46 | 2.70 ± 3.62 | n.s. |
| **MoCa** | 24.25 ± 4.86 | 25.60 ± 2.17 | n.s. |

Demographic and Clinical Data of EA2 Patients with a SARA score ≥ 4 in clinical examination and EA2 patients with SARA score < 4. n.s. = not significant, n = number, yrs = years, hrs = hours, n.d. = non-dominant, sec = seconds, NHPT = Nine-Hole Peg Test, SVV = Subjective Visual Vertical, vHIT = video Head Impulse Test

**Supplementary Table 5**

|  | **EA2 with Medication** | **EA2 without medication** | **Level of significance** |
| --- | --- | --- | --- |
| **Number of patients** | 10 | 8 |  |
| **age (yrs)** | 41.0 ± 11.4 | 42.9 ± 16.4 | n.s. |
| **sex (n female / male)** | 3/7 | 4/4 | n.s. |
| **age at diagnosis** | 10.3 ± 6.5 | 11.4 ± 5.7 | n.s. |
| **disease duration (yrs)** | 30.7 ± 11.9 | 28.9 ±18.1 | n.s. |
| **attacks / month** | 4.6 ± 5.7 | 4.9 ± 8.3 | n.s. |
| **attack duration (hrs)** | 3.6 ± 3.5 | 15.5 ± 34 | n.s. |
| **SARA** | 3.95 ± 2.66 | 4.13 ± 4.48 | n.s. |
| **INAS** | 6.60 ± 3 37 | 4.50 ± 2.39 | n.s. |
| **INAS, occular part** | 4.40 ± 1.96 | 2.38 ± 2.45 | n.s. |
| **DHI** | 44.20 ± 25.38 | 32.25 ± 28.57 | n.s. |
| **CVS** | 5.4 ± 4.72 | 3.37 ± 4.00 | n.s. |
| **MoCa** | 24.40 ± 3.69 | 25.75 ± 3.50 | n.s. |

Demographic and Clinical Data of EA2 Patients with medication (acetazolamide or 4-Aminopyridine) and EA2 patients without medication. n.s. = not significant, n = number, yrs = years, hrs = hours, n.d. = non-dominant, sec = seconds, NHPT = Nine-Hole Peg Test, SVV = Subjective Visual Vertical, vHIT = video Head Impulse Test
